# Correction: Patel et al. Development and Characterization of an In Vitro Cell-Based Assay to Predict Potency of mRNA–LNP-Based Vaccines. *Vaccines* 2023, *11*, 1224

**DOI:** 10.3390/vaccines13020186

**Published:** 2025-02-14

**Authors:** Nisarg Patel, Zach Davis, Carl Hofmann, Josef Vlasak, John W. Loughney, Pete DePhillips, Malini Mukherjee

**Affiliations:** Analytical Research & Development, Merck & Co., Inc., 770 Sumneytown Pike, West Point, PA 19486, USA; npatel886@live.com (N.P.); zpdavis93@gmail.com (Z.D.); carl.hofmann@merck.com (C.H.); josef_vlasak@merck.com (J.V.); john_loughney@merck.com (J.W.L.); pdsld@comcast.net (P.D.)

The authors would like to make the following corrections to this published paper [1].

In the original publication, there was a mistake in Figure 1H,I as these images were accidentally duplicated. There was also a mistake in the cell line name in Figure 1F. The corrected Figure 1 is attached below.

The authors state that the scientific conclusions are unaffected. This correction was approved by the Academic Editor. The original publication has also been updated.

## Figures and Tables

**Figure 1:**
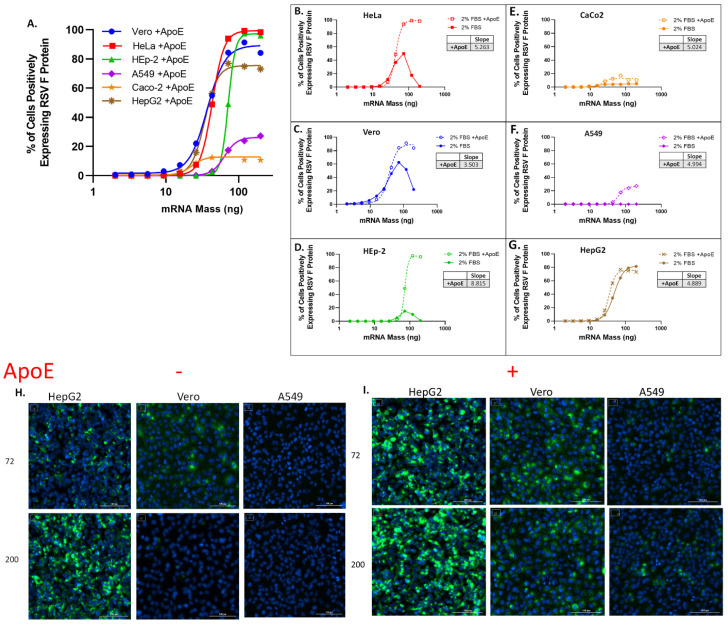
LNP transfection curves across varying cell lines indicate variable levels of transfection, which improve with the addition of ApoE during transfection. (**A**) RSV-F protein expression efficiency in Vero, HeLA, HEp-2, A549, CaCo-2 and HepG2 cells was measured by counting percentage of cells positive for RSV-F protein. All cells were transfected for 16 ± 2 h with titration of LNPs starting with 200 ng dose of mRNA in media with 2% FBS. Data were fit using variable slope-four parameter logistics regression (4-PL) model in GraphPad Prism software (version: 6.0). (**B**–**G**) RSV-F protein expression efficiency following transfection with LNPs in media + 2%FBS, and media + 2%FBS supplemented with ApoE at 4 ug/mL. Hill-slope values [Y = Bottom + (Top − Bottom)/(1 + 10^((LogEC50-X) × Hill-slope)] from 4-PL regression are given for curves obtained with FBS supplemented with ApoE. (**B**) HeLa cells. (**C**) Vero cells. (**D**) Hep-2 cells. (**E**) CaCo-2 cells. (**F**) A549 cells. (**G**) HepG2 cells. (**H**) Representative immunofluorescence images of HepG2, Vero and A549 cells without ApoE and (**I**) with ApoE shown at 72 (top row) and 200 ng/mL (bottom row) of mRNA dose representing the bottom and top of the dose response curve, respectively.
