# Supplementary figures and images for: Development and Characterization of an In Vitro Cell-Based Assay to Predict Potency of mRNA–LNP-Based Vaccines
Source: Vaccines (Basel). 2023 Jul 10;11(7):1224. doi: 10.3390/vaccines11071224 (PMC10383996; doi:10.3390/vaccines11071224)

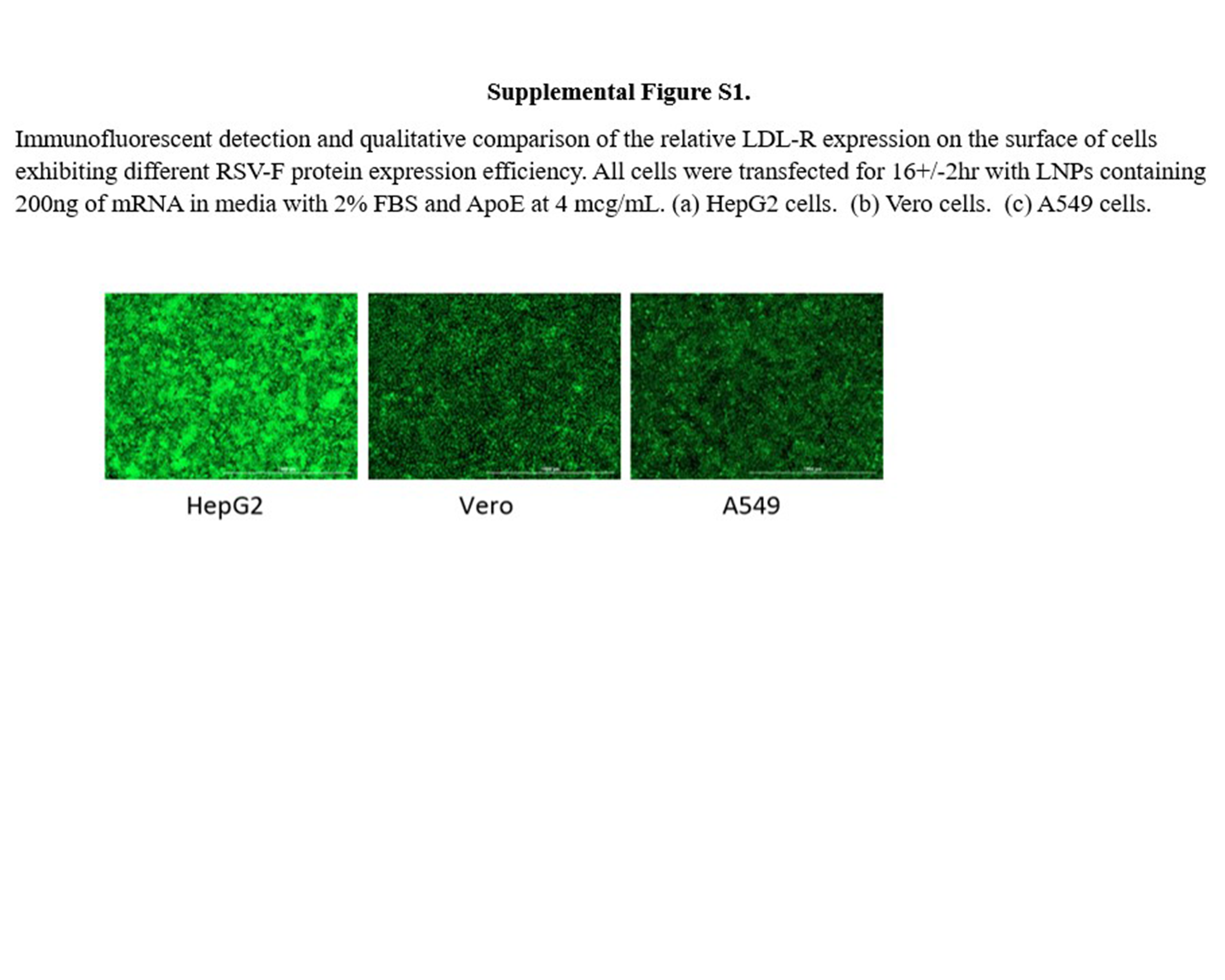

Supplement: Supplementary file 1 [file vaccines-11-01224-s001.zip › vaccines-2461616-supplementary.tif]
